# Creation of a restrictive atrial communication in pulmonary arterial hypertension (PAH): effective palliation of syncope and end-stage heart failure

**DOI:** 10.1177/2045894018776518

**Published:** 2018-04-25

**Authors:** Anna Bauer, Markus Khalil, Dorle Schmidt, Jürgen Bauer, Anoosh Esmaeili, Christian Apitz, Norbert F. Voelkel, Dietmar Schranz

**Affiliations:** 1 Justus Liebig University Clinic Giessen, Hessen Pediatric Heart Center, Giessen, Germany; 2 Johann-Wolfgang Goethe University Clinic, Frankfurt, Germany; 3 Division of Pediatric Cardiology, University Children’s Hospital, Ulm, Germany; 4 Free University Medical Center (VUMC), Amsterdam, The Netherlands

**Keywords:** pulmonary arterial hypertension, PAH, syncope, heart failure, atrial septostomy, restrictive atrial communication

## Abstract

Atrial septostomy (AS) is recommended for pulmonary arterial hypertension (PAH)-associated right ventricular (RV) failure, recurrent syncope, or pulmonary hypertensive crisis (PHC). We aimed to evaluate the feasibility and efficacy of AS to manage PAH from infancy to adulthood. From June 2009 to December 2016, transcatheter atrial communications were created in 11 PAH patients (4 girls/women; median age = 4.3 years; range = 33 days–26 years; median body weight = 14 kg; range = 3–71 kg; NYHA-/Ross class IV; n = 11). PAH was classified as idiopathic (n = 6) or secondary (n = 5). History of syncope was dominant (n = 6); two with patent foramen ovale (PFO) admitted with recurrent PHC, three patients required resuscitation before AS. Three patients had PAH-associated low cardiac output. The average pulmonary arterial pressures (PAP systolic/diastolic) were 101/50 (±34/23); the corresponding systemic arterial pressures (SAP) were 99/54 (±23/11); and the mean ratio of PAPd / SAPd was 0.97 (±0.4). Percutaneous trans-septal puncture was uneventfully performed in nine patients; a PFO was dilated in two patients. There was no procedure-related mortality. The median balloon size was 10 mm (range = 6–14 mm); the mean catheter time was 174.6 ± 48 min; fluoroscopy time was 19.8 (±11) min. Syncope and PHC were successfully treated in all patients. The mean arterial oxygen saturation decreased from 97 ± 2 to 89 ± 11.7. One patient died awaiting lung transplantation, one continues to be listed; two patients received a reverse Potts-shunt, one patient died during follow-up; seven patients are stable with PAH-specific treatment. Percutaneous AS is an effective method palliating PAH-associated syncope, PHCs or right (bi-) ventricular heart failure.

## Introduction

The treatment of severe pulmonary arterial hypertension (PAH) remains challenging, despite improved medical options to address this life-threating condition. Pediatric pulmonary hypertension guidelines^1^ have endorsed atrial septostomy (AS) for patients with right ventricular (RV) failure, recurrent syncope, or pulmonary hypertensive crisis (PHC), but only a few pediatric case series have been reported.^2,3^ In adults, AS is mostly performed as a bridge to lung transplant.^4^ AS is used as an additional therapeutic strategy for PAH-associated right heart failure,^5^ in cases where PAH-specific medical treatment fails^6,7^ and as a palliative, pre-transplant therapy.^8,9^ AS has been demonstrated in case series to improve survival of PAH patients^10^ and to improve outcome, in particular if the approach is combined with PAH-specific treatment.^7^ Lammers et al.^11^ reported that until this date, the worldwide experience with AS was limited to approximately 280 adult and pediatric patients. The rationale for the creation of an atrial septum defect was based on the hypothesis that an iatrogenic atrial right-to-left shunt improves left heart filling, increasing systemic cardiac output and oxygen delivery despite a mild to moderate systemic arterial desaturation. Observations obtained 30 years ago^12^ showed that patients with idiopathic PAH, in whom a foramen ovale was patent, appear to live longer than those without a patent foramen ovale (PFO). Also, patients with an Eisenmenger syndrome related to an atrial septum defect have shown better survival when compared to PAH patients with an intact atrial septum.^13^ Based on this background, we asked the question: should a restrictive atrial communication be prophylactically created in all patients with severe PAH? Here, we report our experience during the last six years in order to evaluate the feasibility and efficacy of percutaneous atrioseptostomy to create a restrictive atrial septum defect (rASD) in the management of PAH-associated syncope, PHCs, or right ventricular heart failure.

## Methods

This retrospective study encompasses 14 percutaneous AS in 11 patients with PAH (four girls/women; median age = 4.3 years; age range = 33 days–26 years; median body weight = 14 kg; range = 3–71 kg). The demographic data are summarized in Table 1. The median time from diagnosis to septostomy was 3 years (range = 1 day–10.3 years). Two catheterizations with AS were performed as high urgency procedures as one component of effective resuscitation. PAH was defined based on hemodynamic assessment in accordance with the World Health Organization (WHO) classification.^8,9^ Six patients had idiopathic PAH, five patients had secondary PAH; two were related to lung parenchymal disease, and two had PAH in the wake of corrective surgery of congenital heart defect. Considering that syncope places patients in NYHA/Ross functional class IV,^1^ all of the patients were in class IV, despite the left ventricular ejection fraction (LVEF) at rest being in an average of 62.3 ± 13.6%; only three patients had a low cardiac output (< 2.5 /L/minxqm). Median arterial pulse-oximetric oxygen saturation at admission was 96% (range = 94–99%). Invasive hemodynamic assessment was performed in ten patients, in five of them immediately before AS; the other patients had a previous catheter investigation. Systolic and diastolic pulmonary arterial pressure (PAP s/d) was 101/50 (±34/23) and the systemic arterial pressures (SAP) 99/53 (±23/11); the mean ratio of PAPd/SAPd was 0.97 (±0.4). The PAP approached or exceeded the SAP in seven patients. The precapillary pressure component was defined by the invasively measured median transpulmonary pressure gradient (difference of mean PAP [mPAP] and mean LAP/wedge pressure) and was 53 mmHg (range = 20–107 mmHg) and the median diastolic pressure gradient was 37 mmHg (range = 13–87 mmHg). The mean superior cava vein oxygen saturation (SvO2) was 59 ± 11.2%. Based on our institutional PH-test protocol,^14,15^ pulmonary endothelial cell functional test was performed by local infusion of two concentrations of acetylcholine. The pulmonary flow reserve (PFR) was determined in seven patients with a baseline average peak velocity (APV) of 18 ± 6 cm/s; the calculated maximal PFR was in a median of 1.7 (range = 1.2–2.8); all tested patients with a history of syncope or PHC showed a degree of residual endothelial cell function. Nine patients, when tested, responded to inhaled NO, aerosolized Iloprost, or to its combination. The average PAPd/SAPd ratio decreased form baseline 0.97 (±0.4) to a maximum of 0.62 (±0.38). Informed written consent was obtained before the procedures from all patients or their parents. Institutional review board approval was obtained for analyzing all retrospective data.

**Table 1.** Characteristics, presentation at admission, and outcome data of patients with PAH palliated with atrioseptostomy.

Patient no.	Diagnosis	Additional diagnosis	Gender	Age (years)	Weight (kg)	Height (cm)	FC (I–IV)*	BNP (pg/mL)	SaO2	TR pre	PAP (sys) (mmHg)	PAP (diast) (mmHg)	TPG (mmHg)	SAP (sys) (mmHG)	SAP (dias) (mmHG)	DPG (mmHG)	PAPd/ SAPd	APV (cm/s)	PFR	Treatment before procedure	Outcome
1^†^	iPAH	PE; resuscitation follow-up, IAS-stenting	M	16.8	64.00	183	IV	360	94	I	145	72	87	110	64	66	1.10	14.00	1.2	S+B+I	D (730 days after P.)
2	iPAH + Syncope	Neurodermitis, convulsions	F	1.87	8.70	81	IV	575	96	I	51	19	20	97	47	13	0.40	–	–	–	A (good long-term)
3	sPAH + Syncope	Surfactant- protein-C deficiency, PHC	F	7.1	17.30	104	IV	46	96	I	146	95	107	116	59	87	1.60	32.00	1,6	S + prednisolon	A no-syncope
4^†^	sPAH + Syncope	dTGA, ASO	M	1.6	12.00	88	IV	222	99	I	87	25	37	111	48	17	0.52	17.00	2,8	S + B + Sp	A
5	iPAH + HF	Moya- Moya-syndrome	M	19.5	71.00	192	IV	1277	94	III	149	79	87	120	73	68	1.10	–	–	S + B + Sp	D
6	iPAH + Syncope		M	4.4	14.00	98	IV	440	99	I	83	46	48	91	52	35	0.88	–	–	S + B + Sp	A
7	PAH + Syncope	MECP2 Duplication syndrome	M	1.24	7.00	68	IV	4049	98	I	94	50	53	62	33	37	1.50	14.00	1.5	–	D
8	sPAH + PHC	Undefined syndrome + PFO + PDA-occl.	M	0.09	3.2	52	IV	–	99	I	56	32	32	60	38	23	0.84	14.00	1.7	S + I + prednisolon	D
9	sPAH + HF	Corrective surgery IAA-Typ A, CoA-stent	M	26	52.00	172	IV	232	94	II–III	106	60	62	80	50	50	1.20	–	–	S + B + Sp + PGIiv	A
10	sPAH + Syncope	Nodular heterotopy (filaminA) PDA-occ	F	23.6	53.00	160	IV	48	98	I	72	26	33	135	64	16	0.42	16.00	2.3	–	A
11^†^	PAH + PHC	PFO /RV-failure (17% RVEF)	F	0.4	7.00	63	IV	1919	95	I–II	119	50	61	103	53	38	0,94	17	1,9	–	A
	Mean			9.33	28	115		917	96.55	–	101	50	57	99	53	41	0.97	17.71	1.86		
	SD (±)			9.6	24.85	49.55		1186	2.02	–	33.77	23.26	25.95	22.61	11.17	23	0.40	5.97	0.50		
	Min			0.09	3.2	52		46	94.00	I	51	19	20	60	33	13	0.40	14	1.2		
	Max			26	71	192		4049	99.00	III	149	95	107	135	73	87	1.60	32	2.8		
	Median			4.4	14	98		400	96.00	I	94	50	53	103	52	37	1.02	16	1.7		

* NYHA/Ross categories.

^†^patients with pulmonary hypertensive crisis, only ballooning of PFO

A, alive; APV, average peak velocity; ASO, arterial switch operation; B, Bosentan; I, Iloprost; BNP, brain natriuretic peptide; CoA, Coarctation; D, death; dias, diastolic; DPG, diastolic pressure gradient; dTGA, dextro-transposition of the great arteries; EF, ejection fraction; FC, functional class; IAA, interrupted aortic arch; IAS, interatrial septum; IPAH, idiopathic pulmonary arterial hypertension; HF, heart failure; MECP2, reversible Rett-syndrome; P, procedure; PAP, pulmonary arterial pressure; PAPd/SAPd, pulmonary to systemic diastolic pressure ratio; PDA, patent ductus arteriosus; PE, pericardial effusion; PFO, patent foramen ovale; PFR, pulmonary flow reserve; PGI, Prostacyclin (Epoprostenol); PHC, pulmonary hypertensive crisis; RV, right ventricle; S, Sildenafil; SaO2, arterial oxygen saturation; SAP, systemic arterial pressure; SD, standard deviation; Sp, Spironolactone; Sys, systolic; TPG, transpulmonary pressure gradient; TR, tricuspid regurgitation.

Statistical data were calculated with SPSS (IBM). Descriptive data are presented as mean and standard deviation (mean ± SD) or median and range, as appropriate. Significance was calculated using the Wilcoxon-test for non-paired samples. A *P* value ≤ 0.05 was considered statistically significant. Continuous variables are presented as mean and standard deviation (mean ± SD) or median and range as appropriate. All variables were analyzed only if both pre- and post-intervention data were available.

### Interventional technique

**Fig. 1.**
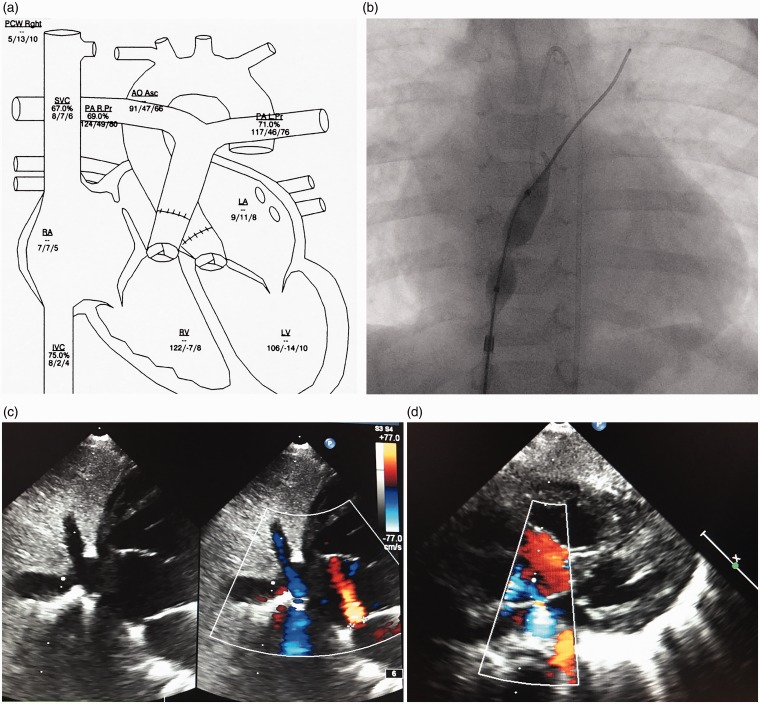
Data of a patient 4 with a history of life-threatening syncope. (a) The catheter heart cartoon; the measured PAP data obtained to different time intervals shown not only a difference between the left and right pulmonary artery but more important to the PAP data depots in Table 1, measured just before trans-septal puncture in deep sedation. The PAPs fixed on the cartoon shows a suprasystemic pressure level. By this example, the hyper-reactive pulmonary artery system can and should be demonstrated. The venous oxygen saturation of the SVC of 67% and mixed venous oxygen saturation of 69% represents a normal cardiac output in the catheter laboratory during the procedure with analgo-sedation. The LAP, measured just after trans-septal puncture and after balloon dilatation of the atrial septum, shows that the LAP is still higher than the RAP at rest demonstrating a restrictive atrial septum defect. (b) High-pressure balloon (Power-Flex) still not fully inflated within the atrial septum, starting with the first inflation immediately after trans-septal puncture; (c) 2D color echocardiography of the created restrictive atrial septum defect with a small left-to-right shunt at rest; (d) right-to-left shunt (blue color) on demand of the same patient. AoAsc, ascending aorta; IVC, inferior caval vein; LA, left atrium; LV, left ventricle; PALPr, left pulmonary artery pressure; PARPr, right pulmonary artery pressure; PCWP, pulmonary capillary wedge pressure; RV, right ventricle.

All procedures were performed under fluoroscopy guidance. Echocardiography immediately before the procedure was performed to rule out a small, volume-unloaded left atrium, which increases the risk of the procedure. Local anesthesia was performed in all patients; young children received analgesia-sedation and only one procedure (patient 1) was performed under anesthesia due to resuscitation conditions, when a stent had been implanted within the interatrial septum (IAS). The procedure and results are summarized in Table 2 and as an example in Fig. 1. Infants and young children received diazepam or midazolam combined with ketamine as single and repeated intravenous bolus, adolescents or adults only by request, pethidine as a single intravenous dose and/or propofol as a continuous infusion in a dosage of 1–2 mg/kgxh. Femoral vein access was secured with an 8-F or 10-F Terumo short sheath, while the two youngest patients received a 6-F sheath. For diagnostic purposes, femoral arterial access was secured with a 4-F or 5-F sheath. In stable patients, a complete hemodynamic assessment was performed before the intervention, in critically ill patients, only the intervention was performed. In five patients, MRI was performed before or as a hybrid imaging procedure with subsequent heart catheterization. In younger patients, especially in infants, this was carried out during a single episode of sedation. Trans-septal puncture was performed in nine patients using a Brockenbrough technique (Table 2) with a needle length of 56 cm or 71 cm (Cook Medical). In children, the needle was loaded into a 6-F, 48-cm-long Check-Flow performer® Introducer (Cook Medical); in adolescents and adults an 8-F Mullins sheath (Cook Medical) was used. To ensure safety during the manipulation, the long sheath was advanced through the already-placed 2-F larger short sheath in all but the two youngest patients. Under biplane fluoroscopy guidance (anteroposterior [AP] and 90° lateral view the long sheath was first advanced over a 0.035-inch guide-wire placed in the superior caval vein (SVC). After the Cook long sheath was placed in the SVC, the wire was removed. The trans-septal needle filled with contrast medium via a 5-mL Luer-Lock syringe was then smoothly advanced 1–2 mm beyond the tip of the mandril. The needle-sheath ensemble was pulled back from the SVC into the right atrium (RA) during continuous ejection of contrast medium through the needle. For an interatrial septum (IAS) puncture, the tip of the puncture ensemble was directed posteriorly on the 90° lateral view and between the 3 and 4 o’clock positions on the AP view. On contact with the fossa ovalis, the needle was advanced to inject contrast medium into the IAS, then advanced to the LA by further continuous injection of contrast medium. After entering the LA, the Cook sheath was advanced across the IAS and the needle replaced with a 0.035-inch guide-wire. In small children, when direct passage of the long sheath appeared to be risky, or the IAS appeared to be very thick, a coronary guide-wire (“Whisper,” Abbott) was used for the advancement through the needle into the LA to allow for balloon pre-dilatation of the IAS. Removal of the sharp needle together with the mandril was carried out cautiously, to avoid guide-wire shearing. By ballooning the IAS with a coronary or peripheral balloon (3–6 mm width and 20–30 mm length), positioning of the 6-F long sheath into the LA was achieved by advancing the sheath over the deflating balloon. This trans-septal puncture technique is also the institutional approach for neonates with hypoplastic left heart syndrome and intact atrial septum.^16,17^ In older children and in adults, a stiff 0.035-inch Amplatzer® guide-wire was preferentially positioned in the left upper pulmonary vein. IAS balloon dilatation was then performed with high-pressure balloons; depending on the patient’s size, Powerflex® or ZII-PFM® balloons with a length of 20–40 mm were used. The maximal balloon diameter was chosen depending on the desired size of the atrial communication. In all patients, the goal was to achieve a restrictive atrial communication; therefore, a somatic size-dependent communication of 4–10 mm was created. Based on our long-term experience,^18^ static ballooning of the IAS was in part performed by sequential dilatation using balloon diameters up to a maximum of 14 mm resulting in an arterial communication of up to 8 mm Powerflex® 12 × 30 mm balloons or in adolescents and adult patients, ZII (PFM) balloons (14 × 20/40 mm) were predominantly used as the final balloon diameter with an average balloon diameter of 11 mm (range = 6–14 mm) (Table 2). Indentation by the IAS on the waist of the inflated balloon, followed by disappearance of the waist after full inflation, was identified in all procedures (Fig. 1b). If recoil was observed during deflation, repetition of inflation up to the rated burst pressures (8–12 atm) was performed. At the end of the procedure, full or partial hemodynamic data were obtained and the created atrial communication was evaluated by transthoracic echocardiography. Persistent foramen ovale dilatation without trans-septal needle puncture was performed in two patients. The same balloon material and technique were used as described. In one patient (patient 1), a Genesis premounted stent (10 × 19 mm) was placed into the IAS during a second catheterization, the balloon was smoothly inflated such that a residual hourglass shape with a waist diameter of 8 mm was achieved, creating a restrictive atrial communication. Procedural success was defined as a successful creation of a patent, but a restrictive atrial communication. Restriction was defined by echocardiography and only in part by measuring a residual RAP/LAP or LAP/RAP gradient. Considering the experience with congenital ASDs, an atrial communication is in any case restrictive, when the diameter of the atrial communication is < 25% of the total septum length. In this context, gradual balloon dilatation of the IAS was favored in some of the patients, in order not to create an unrestrictive atrial communication. The decision for re-dilatation or stent placement of the IAS during a later follow-up was made depending on the clinical condition of the patients and by echocardiographic evidence of recoil or almost closure of the created atrial septum defect.

**Table 2.** Procedure-related data before and after gradual balloon dilatation of the atrial septum, in nine patients after trans-septal puncture by the Brockenbrough technique.

Patient no.	AS needle (Yes/No)	Procedure (min)	Fluoroscopy (min) + VAT	Balloon size max. (mm)	RAP- pre (mmHg)	RAP-post (mmHg)	PCWP/LAP- pre (mmHg)	LAP-post (mmHg)	SaO2 - post (%)	R/L-shunt at rest (Yes/No/Bi)	Subsequent treatment	AS clinical effect
1	Yes	128	14	12	13	11	6	8	90	Yes	S, SP, B	Short; Stent*
2	Yes	91	10	8	3	2	3	3	98	No	S, A	No syncope
3	Yes	281	46	12	6	7	8	5	92	Bi	S, SP, B, I + Prd	Resusc in Cath.
4	Yes	172	11	10	5	6	5	8	97	No	S, SP, A	No syncope
5	Yes	178	27	14	23	21	14	14	82	Yes	S, SP, A, B	Transient, Potts-S
6	Yes	176	19	8	8	11	4	9	93	Bi	S, SP, B	No syncope
7	Yes	196	16	8	12	12	12	12	95	Bi	S, SP, I	No syncope
8	No	155	15	8	3	3	3	6	90	Bi	S, SP, I, A	No PHC
9	Yes	–	–	14	21	–	–	20	55	Yes	S, SP, I, B	ASD-Occl 12 mm
10	Yes	210	31	10	4	4	7	7	90	Bi	S, SP, A, Ambrisentan	No syncope
11	No	159	9	6	6	5	9	10	99	No	S, A	No PHC
Mean		174.6	19.8	10	9.5	8.2 7.1		9.3	89.2			
SD (+/-)		47.8	11	2.6	6.7	5.4 3.5		4.5	11.7			
Min		91	9	6	3	2 3		3	55			
Max		281	46	14	23	21 14		20	99			
Median		174	15	10	6	6.5 6.5		8	92			

* IAS stenting

A, amlodipine; AS, atrioseptostomy; ASD, atrial septum defect; B, Bosentan; Bi, bidirectional shunt; I, Iloprost; LAP, left atrial pressure; Occl, occluder; PCWP, pulmonary capillary wedge pressure; PHC, pulmonary hypertensive crisis; Potts-S, Potts-shunt; Prd, prednisolone; RAP, right atrial pressure; Resusc, resuscitation; R/L, right/left shunt; S, sildenafil; SaO2, arterial oxygen saturation; Sp, spironolactone;

## Results

A restrictive atrial communication was generated in all patients. The size of the communication in all of the patients was < 9 mm; the median size was 5 mm (range = 4–8 mm) as assessed by two-dimensional (2D) and color flow echocardiography immediately after the procedure. Percutaneous trans-septal approach was performed in the median of three days (range = 1–11 days) after admission. The median procedural length (including hemodynamic assessments) was 174 min (range = 91–281 min); median fluoroscopy time was 15 min (range = 9–46 min) (Table 2). There was no procedural death and there were no complications, with the exception of one patient (patient 9). In this patient, AS was uneventfully performed despite and because of unstable hemodynamics immediately following surgical Potts-shunt placement. Considering a still extreme high (20 mmHg) right atrial pressure (RAP), we performed an atrial communication. However, the physiopathology of AS together with the reverse Potts-shunt induced a too severe hypoxemia (< 55% SaO2). Utilizing an Amplatzer 12 mm ASD occluder, the generated atrial defect was immediately closed. In addition to the described patient (patient 9), six patients showed a decrease of SaO2 following the creation of an atrial communication; only one (patient 5) responded with a SaO2 < 90%. The mean oxygen saturation (n = 10) decreased by 7.3 percentage points (*P* < 0.05). Three patients showed (by TTE) a right-to-left shunt, five showed a bidirectional shunt, and three patients showed a left-to-right shunt at rest. The immediate obtained averaged data of RAP and left atrial pressure (LAP) did not significantly decrease (9.5 ± 6.7 to 8.2 ± 5.4) or increase (7.1 ± 3.5 to 9.3 ± 4.5), respectively. None of the patients subsequently experienced a syncope and all improved symptomatically with a significant (*P* < 0.01) decrease in functional class (median shift I); the improvement of NYHA/Ross functional class from a median of class IV to III correlated with serum brain natriuretic peptide (BNP) values; the median serum BNP decreased from 916 pg/mL (range = 46–4049 pg/mL to 539 pg/mL (range = 31–1848 pg/mL) (*P* < 0.05) between admission and discharge. The PAH-specific medication after AS and before discharge was changed in patients with syncope and PHCs to include the PDE-5-inhibitor, sildenafil, and the calcium-antagonist, amlodipine; none of the patients had been admitted with calcium-antagonist treatment; yet, after creation of the restrictive atrial communication, six were discharged home with additional long-acting amlodipine treatment (Table 2).

### Follow-up

All patients had an in-hospital follow-up of a median of eight days (range = 1–94 days); the outcome was followed in 11 patients over a median time of 532 days (range = 13–2082 days) after the procedure. Following the primary intervention, three patients received a second intervention 28, 50, and 238 days after the first procedure. In one patient (patient 4), the atrial septum was manipulated two times. Re-interventions were clinically and echocardiographically indicated for re-dilatation of a patent but recoiling and increasingly restrictive atrial communications. Patient 1 remained stable for 730 days after the atrial septum stent placement but died on the waiting list for lung transplantation. Patient 5, with dominant right- but bi-ventricular heart failure, had only a short clinical improvement and a trans-catheter Potts-shunt was created allowing discharge home. However, the patient had died on follow-up.^15^ Two patients died after 452 and 92 days, respectively. After a median follow-up of 1.8 years (range = 13 days–5.7 years), seven of the 11 patients are alive. One patient (patient 11), admitted at the age of five months, normalized both her right heart function and pulmonary arterial pressures; the atrial communication was closed two years later by an Amplatzer ASD 8-mm device.

## Discussion

Although we present data on a relatively small number of patients, the present study confirms that generation of a restrictive atrial communication in PAH patients is a safe and effective approach, even as a high urgency procedure performed under resuscitation conditions. Improved techniques and materials allow performing AS at any age, even in infancy. The generation of an atrial communication might be the most effective approach in critically ill patients, but it remains a palliation.^2–7^ At our institution, atrioseptostomy with the technique described here has been established for more than two decades as part of the “Giessen Hybrid” approach for treating newborns with hypoplastic left heart syndrome.^16,17^ Based on this long-term and age-independent experience, we utilize AS as an early treatment in infants, children, and young adults with idiopathic and secondary PAH-associated syncope, PHC, or cardiac failure. However, weighing the risks and benefits of AS has to be considered in context of the institutional experience. Additionally, we want to emphasize that the pathophysiology of a syncope is quite different in patients with a sustained right heart failure and those of young PAH patients with repetitive life-fearing attacks often ending in severe tonic convulsions, as even observed in some of our patients (patients 2, 4, and 6). However, in both hemodynamic conditions, the generated communication needs to be restrictive, avoiding a too large-left-to-right shunt, which would favor pulmonary arterial shear stress, or in case of right heart failure, a too large right-to-left shunt already at rest with a consecutive excessive hypoxemia. In the young patients reported here, generation of a restrictive atrial septum defect prevented further syncope and PHC. According to adult PAH guidelines, AS is usually recommended as a palliative therapy for PAH in patients with end-stage right heart failure, who received maximal medical therapy or as a bridge to lung transplantation.^1,8,9^ Regarding these guidelines, it is of our high interest to report that in young patients with a history of severe syncope or PHC, left ventricular cardiac output, mixed venous oxygen saturation, and RAP might be normal at rest, and pulmonary hypertension also under the systemic blood pressure level (Fig. 1a). In addition, as shown in some of our patients, RAPs might be low only (2–3 mmHg) or maximal (4–5 mmHg) rather related to a low volume status than clinical signs of right heart failure . However, life-threatening and repetitive syncope are nevertheless being avoided. In such a scenario, AS is utilized to treat an acute, life-threatening syncope, which is especially observed in young PAH patients with a highly reactive pulmonary precapillary vasculature. Data of the PFR support these observations of a hyper-reactive pulmonary precapillary vessel, respecting not only a residual but even exceeding endothelial function.^19^ Therefore, based on our PAH test protocol,^14,15^ which has included the endothelial function test for two decades, an additional therapeutic goal is to achieve a sustained pulmonary vasodilation with avoiding episodes of pulmonary vascular hyper-reactivity by supporting the endogenous vasodilative properties. Cardiac syncope, defined as an acute loss of consciousness, is related to a sudden cessation of cerebral blood flow. The pathophysiology of such a PAH-related syncope is characterized by its limits to increase acutely the pulmonary blood flow despite a well-functioning and sufficiently working right ventricle with adequately functioning right-heart valves. The left ventricle “pumps dry”^3^ leading to reduced organ perfusion which results in clinical symptoms of chest pain and, especially in some young patients, epileptic convulsions. Incorrect diagnosis leads to long-term antiepileptic drug treatment because of epileptic-like convulsions; a direct consequence of excitement’s or other stimuli-induced imbalance of systemic oxygen delivery and oxygen consumption. During the syncope-free time, most young patients remain clinically stable because of missing congestive symptoms and a normal systemic cardiac output at rest. Therefore, creation of an interatrial-communication resulted in a dominant left-to-right or bi-directional shunt at rest, depending on the ratio of the LV and RV compliance (Fig. 1c, d). Patients with a persistent foramen ovale or an artificial atrial communication with a too restrictive, and therefore insufficient, shunt supply might clinically present with a PHC; from our perspective, the indication to enlarge the atrial communication by balloon dilatation. Despite the limitations inherent in our cases series, the described results to treat syncope and PHC are promising.

In other scenarios—more often observed in adolescent and adult patients with insufficient RV performance and consecutive systemic vein congestion—AS is utilized to improve chronic systemic low cardiac output on expense of a slightly induced chronic hypoxemia. In our small series, the latter scenario was observed in three adult patients. Two of them finally underwent a surgical Potts-shunt. Detrimental ventricular–ventricular interactions, which reduce LV filling and severely affect LV mechanics, can likely explain an ineffective and too late performed restrictive atrial communication. In this scenario, the creation of a surgical or interventional right-to-left shunting Potts-shunt might be indicated before lung transplantation.^19,22,23^ In a further adult patient (patient 1), AS stabilized the clinical condition, but the created atrial communication became too restrictive after a two-month follow-up. He was referred back in cardiogenic shock; during resuscitation, stenting of the atrial septum together with medical interventions saved his life and allowed the patient to be listed for lung transplant for over two years. However, this case also shows the potential problem of exclusive static balloon dilatation for creating an atrial communication. Considering the variable anatomical structure of the atrial septum, the long-term efficacy of the created communication solely performed by balloon dilatation is unpredictable. Device-based atrial communications might solve this problem in future.

## Study limitations

First, we have to consider that none of the PAH therapies/ treatments currently available are curative and PAH remains a life-threatening condition. Second, creation of a restrictive atrial communication is a palliative approach. Our report is limited because of its retrospective design and a partially incomplete dataset. Follow-up imaging (i.e. MRI data) was not available to quantitate the impact of the interventions in addition to the assessment of the clinical effects and we were unable to present all detailed hemodynamic measurements. Our cohort is small and heterogeneous with respect to age, body size, and underlying causes of PAH.

## Conclusion

Our results documenting the generation of a restrictive atrial communication in a small cohort of children and young adults with life-threatening PAH are still encouraging. The creation of an atrial defect using a trans-septal needle technique followed by gradual ballooning allows the generation of a tailored restrictive communication. Such a procedure should be considered as a safe, cost-effective therapeutic palliation in the setting of PAH-related syncope and PHC. Palliation of choice of syncope as a consequence of a highly reactive precapillary pulmonary vasculature might be the combination of a restrictive atrial communication with endothelium targeted PAH-specific drugs, especially in young children. It is worth emphasizing that the AS must not necessarily induce cyanosis with a right-to-left shunt at rest in order to be effective in young PAH patients with syncope or PHC; the latter is indeed the case in patients with congestive right heart failure. In the near future, device-based interatrial flow regulators with a defined diameter < 10 mm are becoming available having the potential to avoid spontaneous closure and re-interventions.^24^ However, the described procedure of AS should not be limited by the number of interventionists with access to such devices, rather these life-saving interventions need to be available to all patients who may benefit.
